# Clarifying how to deploy the public interest criterion in consent waivers for health data and tissue research

**DOI:** 10.1186/s12910-020-00467-5

**Published:** 2020-03-20

**Authors:** G. Owen Schaefer, Graeme Laurie, Sumytra Menon, Alastair V. Campbell, Teck Chuan Voo

**Affiliations:** 1 Centre for Biomedical Ethics, National University of Singapore, Yong Loo Lin School of Medicine, Block MD11, Clinical Research Centre, #02-03, 10 Medical Drive, Singapore, 117597 Singapore; 2 School of Law, University of Edinburgh, Old College, South Bridge, Edinburgh, EH8 9YL UK

**Keywords:** Public interest, Public good, Consent waivers, Ethics review, Research ethics

## Abstract

**Background:**

Several jurisdictions, including Singapore, Australia, New Zealand and most recently Ireland, have a public interest or public good criterion for granting waivers of consent in biomedical research using secondary health data or tissue. However, the concept of the public interest is not well defined in this context, which creates difficulties for institutions, institutional review boards (IRBs) and regulators trying to implement the criterion.

**Main text:**

This paper clarifies how the public interest criterion can be defensibly deployed. We first explain the ethical basis for requiring waivers to only be granted to studies meeting the public interest criterion, then explore how further criteria may be set to determine the extent to which a given study can legitimately claim to be in the public interest. We propose an approach that does not attempt to measure magnitude of benefit directly, but rather takes into account metrics that are more straightforward to apply. To ensure consistent and justifiable interpretation, research institutions and IRBs should also incorporate procedural features such as transparency and public engagement in determining which studies satisfy the public interest requirement.

**Conclusion:**

The requirement of public interest for consent waivers in secondary biomedical research should be guided by well-defined criteria for systematic evaluation. Such a criteria and its application need to be periodically subject to intra-committee and intra-institution review, reflection, deliberation and amendment.

## Background

There is a small but growing number of jurisdictions that have a public interest or equivalent criterion – public good or public benefit – for granting consent waivers for secondary biomedical research involving personal data^1^ and/or human tissue. For example, New Zealand guidelines on waivers require that “public benefit outweighs public interest in privacy” [1], while Australian guidelines requires that the public interest in the research substantially outweighs the public interest in privacy [2]. In 2015, Singapore enacted a law requiring, among other things, that consent waivers only be granted for “human biomedical research or health information research [that] would reasonably be considered to contribute to the greater public good” [3]. And in 2018, in updating its laws to comply with the European Union’s General Data Protection Regulation, Irish regulations stipulated that waivers only be granted when “the public interest in carrying out the health research significantly outweighs the public interest in requiring the explicit consent of the data subject” [4].

In all these cases, the public interest criterion is not the sole criterion for granting a waiver of consent. Other criteria such as minimal risk are used together with public interest to determine whether consent waiver should be granted. Institutional review boards (IRBs)^2^ tasked with approving or rejecting these waivers may, however, lack formal guidance on how to interpret and implement the public interest criterion. This is a concern for at least two important reasons. Public interest is an amorphous and mutable concept whose interpretation depends on the circumstances of the situation [5]. This flexibility notwithstanding, its rigorous application as a criterion for consent waiver necessitates an appreciation of the ethical import and reason for the criterion. Unclear understandings could cause confusion among researchers applying for the waivers, or dissuade research that could only proceed with a waiver. Moreover, they may lead to IRBs setting a low ethical bar for the criterion which distorts its import and undermines public trust in research, or an overly high bar that hinders its reasonable application and in turn the efficiency of health research regulation.

Second, where there is a lack of clear guidance, different IRBs may apply the standard quite differently – especially if waivers may be granted under expedited or exempt review, with only a chairperson and/or a delegated member assessing its appropriateness. Inconsistency in application of waivers could lead to the same research project being granted a waiver by one IRB, and rejected by another, introducing a degree of arbitrariness and unfairness into the review process that should be avoided. While inconsistency is a more general phenomenon in IRB review [6–9], some of which may be attributed to reasonable variability in institutional context, the addition of a public interest criterion is problematic if it exacerbates those inconsistencies.

Thus, the risk of lack of clarity and consistency in the application of the public interest criterion is problematic, and this is particularly so given the gravity of overriding the individual autonomy interest of citizens in knowing the disposition of their data and controlling access to and use of it via consent. In view of the increasing number of jurisdictions and institutions around the globe that now rely on the criterion to justify human health research without consent, it is timely and important to consider how it may be defensibly deployed. Accordingly, this paper interrogates the notion of the public interest in consent waivers for secondary research on personal data or human tissue, and offers some suggestions on how to apply it in practice with robust ethical legitimacy. To ensure that IRB’s judgments are more consistent, objective, and justifiable, we propose an approach that is guided by substantive and procedural justifications. Our proposal should be of interest to researchers, ethicists and IRBs in jurisdictions that implement or enforce a public interest requirement on consent waivers. And while we focus on consent waivers because of the particular challenges arising in that context, this analysis may also be of use for evaluations of public interest in research in other contexts. For example, our suggestions on procedural mechanisms for assessing public interest may also be applicable to dispersal of tissue stored in biobanks with the consent of patients and under the expectation that such dispersal will advance public interests. Elsewhere, one of us has argued that ‘public interest’ is a preferable term to use among the various concepts that are deployed in this debate^3^. ‘Public good’ is a term of art in economics referring to non-rivalrous and non-excludable resources, while ‘public benefit’ is too narrow by excluding relevant considerations of justice. For the purpose of this paper, we take the term ‘public interest’ to be synonymous with ‘public good’ and ‘public benefit’, with the understanding that whatever term is used, it needs to be juxtaposed against the other public interests that are in play – notably, individual autonomy and privacy – in determining whether consent waiver for secondary research serves and benefits the public overall.

## Main text

### Ethical relevance of the public interest criterion

The present discussion concerns consent waivers for secondary research on personal data or human tissue – that is, new research using data or tissue previously gathered as part of clinical care, a distinct research project, or in another context.

Any use of data or tissue would need compelling ethical justification because personal data and human tissue hold potentially sensitive information in which the sources of the data/tissue, i.e. citizens, have an autonomy and/or privacy interest. In contemporary health research regulation, such justifications take one of roughly four forms: authorisation, anonymisation, subject benefit and public interest. (See Table 1).

**Table 1:** Four forms of justification for use of personal data/tissue

Subject authorisation	Data subjects may consent to the use of data or tissue for a specified purpose (specific consent), or else agree to a wider range of uses, subject to a trustworthy system of oversight (broad consent) [10, 11]. Either way, ethical justification for the use comes in part from subjects being given the opportunity to exercise their autonomy in deciding whether the stated use is permissible [12, 13].
Anonymisation	Regulatory regimes typically exclude anonymised tissue and data from consent requirements, in part on the grounds that if it is sufficiently impracticable for someone to be able to re-identify someone in a dataset, the individual risk profile of using the data will be exceedingly low [7, 14]. Interestingly, Singapore’s Human Biomedical Research Act is the first in the world to require consent even for anonymised secondary tissue research, so this justification would not be available there.
Subject benefit	In some cases it may not be possible to obtain consent, but the research would directly benefit the subject. In this case, the benefit to the subject would have to be deemed as weightier than any privacy/autonomy interest the subject would have in limiting the use of their data or tissue. Some forms of emergency research could fall in this category, though it is unlikely to be applicable to secondary data/tissue studies under consideration here.
Public interest	In other cases, data cannot be anonymised,^a^ it would not be practicable to obtain consent, and the relevant use is not of direct benefit to the individual. Still, a waiver could be justified on the grounds it contributes to the public interest. Here, that contribution to the public interest would similarly have to be sufficient to override any privacy/autonomy interest individuals may have in limiting the use of their data or tissue.

^a^Either because the data is only useful in identifiable format, or because anonymisation is not feasible. The latter category is becoming increasingly true of datasets previously thought to be anonymised [15, 16]

It is commonplace to rely on the first two forms, encompassing the so-called ‘consent or anonymise’ approach. In those cases, either participant authorisation is sought, or data/tissue is anonymised to minimise risks to the participant. In other instances, as with some emergency research, the third form of subject benefit might be used to justify the research. But sometimes consent is not practicable, research requires the use of identifiable data, and subjects would not directly benefit.

Furthermore, in many jurisdictions, such as the United States and Singapore, health research regulation allows for waivers of consent whereby the ethical imperative to seek authorisation from the data/tissue subject is bypassed when sufficiently good ethical reasons are given to do so. Often, this is done in the name of the public interest.

In those cases where the other three justifications are not applicable, ‘public interest’ will be doing the moral work of justifying the permissibility of the secondary research. This requirement is similar to concerns in other areas of governance: individual, private interests may be overridden (subject to constraints) when the public interest is sufficiently great [17]. As mentioned, research regulatory regimes that include a public interest criterion do not treat this threshold as sufficient in itself to grant a waiver; other requirements such as minimal risk, the necessity of using identifiable information, or impracticality in obtaining individual consent would apply. However, where public interest is deployed it is viewed as a necessary condition for granting a waiver, insofar as good reason needs to be given why it is permissible to override the privacy or autonomy interests of subjects in maintaining control over their data or tissue.

Though public interest might exist on a continuum, with some studies advancing it more than others, the public interest *criterion* is binary – either a study meets the requirement or it does not by meeting a certain threshold along that continuum. Because of this, it becomes all the more important to understand where this bright line might be drawn and how it can be justified.

### Working definition of public interest

With this understanding of the justificatory role of ‘public interest’ in consent waivers, we can now explore how we might define the concept in practice in ways that give full effect to its ethical basis, while also providing some means to deliver a degree of consistency in decision-making.

One immediate worry about a public interest criterion is that it may be redundant. It is generally accepted that all research must demonstrate some degree of potential social value of its results in order to be ethically justified – as reflected, for example, in the most recent revisions to the Council for International Organizations of Medical Sciences (CIOMS) guidelines on human subjects research [18]. Without a social value requirement on all research, there would be little reason to impose risks on subjects, undertake administrative burdens of oversight and expend infrastructural resources required. But if ‘public interest’ were simply interpreted as a requirement of all research akin to a synonym for social value, the public interest criterion of consent waivers would be trivial: a technical requirement that has no additional practical or ethical import.

Yet, a trivial understanding would only make sense if it played a trivial role. As it is used in health research regulation, establishing the public interest produced by a given study is quite critical to providing *additional* and thorough ethical justification of a waiver of consent. For example, with respect to the Singapore context, the Ministry of Health explained (during stakeholder communication) that the greater public good criterion in the 2015 law would not be routinely met by secondary data and tissue research as currently practiced. That is, it must meet a higher bar of public good (hence ‘*greater* public good’) than the minimum expected of all research. This is reflected in the CIOMS guidelines’ recommendations concerning waivers of informed consent. In that context, the guidelines clarify that the research must have “important social value” (p. 7) to meet the waiver criterion, and yet the qualifier of ‘important’ is left unspecified [18]. In other words, while all research must demonstrate at least the promise of some social value, we contend that appeals to public interest or the public good go further requiring more specific and arguably more arduous standards to justify a waiver of consent.^4^

For a non-redundant, non-trivial understanding of public interest in consent waivers in the context of biomedical research, we will propose the use of the following definition:

Contribution to the public interest: Substantial expected advancement of the health-related interests of members of a group whose interests are, or should be, of particular concern to the society in question.

Several features of this definition are worth highlighting. ‘Substantial’ will be crucial, distinguishing it from a mere social value criterion. This may relate to the magnitude of the benefit of the research, as well as the number of individuals potentially affected – though how to adjudicate an appropriate threshold will be revisited in our analysis below. ‘Expected’ acknowledges that, ex ante, we cannot know whether a study will succeed, so assessment of success would have to be tempered by likelihood of success. The difficulty in assessing the magnitude and probability of the good generated by research motivates, in part, our analysis below. ‘Interests’ is understood broadly, to include not just material or physical well-being but also considerations of justice, such as equitable distribution of resources.

‘Groups of concern’ allows for research whose benefits are targeted to a particular subpopulation, such as patients affected by a particular disease. But, why not society (or humanity) at large? The justificatory work of the public interest discussed above does not require that *all* of society benefit. Some research may indeed benefit all of society and humanity, but others will only benefit a subset of society, such as children, but nevertheless be of enough importance to justify a waiver.^5^ Why not then specify more simply that it should benefit some group or subgroup? This would make the definition too inclusive – e.g., research that only benefitted the profit margins of a study sponsor. So the definition is made relative to groups whose interests are of particular concerned to advance via the research. An example might be rare disease groups who, by definition, only represent a small percentage of the population as a whole, but whose interests in proper diagnosis and treatment can only be advanced by supporting specific research into their condition. Furthermore, we must distinguish between direct and indirect benefit. While it is the case that the only the rare disease group will directly benefit from the research, it might also be true that wider society indirectly benefits from the generalisable knowledge that is produced from the research findings; for example, by creating a new research database for yet further future research use.

Finally, ‘are or should be’ is added to include marginalised groups whose interests are unjustly ignored by society at large; such research may contribute to the public interest, even if most members of the public wrongly fail to recognise it. For example, in Europe there are widespread negative attitudes towards the Roma people [19], which has led to substantial health disparities and a commensurate need for greater attention from health researchers [20].

### Setting the bar

The question, then, naturally turns to how contribution to the public interest should be determined. This matter is somewhat related to another issue with which IRBs must regularly grapple – whether a given study has an acceptable risk-benefit ratio, taking into consideration both individual subject and public benefit [21]. And as with this last question, there is no strict formula or universal checklist that can be applied to determine the appropriate bar.

This analysis encompasses two levels of inquiry: the *substantive* question of whether a given study is in fact in the public interest; the *procedural* issue of how systems (local, institutional or even national) should be designed to legitimately and reliably evaluate the public interest criterion. Our procedural analysis will highlight the features needed at the outset of setting up policies or practices around the public interest criterion, as well as the further need for long-term reflexivity on those policies or practices by relevant bodies – making them constantly subject to revision, with active procedures in place to facilitate this.

#### Criteria-based approaches to the substantive question

An attractive approach would be for institutions and IRBs to establish a set of evaluative criteria for meeting the public interest requirement. Upon submission of a protocol for review, researchers would answer a series of questions (e.g., related to impact, relevance to health priorities, likelihood of success), which would then be assessed by the IRB. This allows for careful attention to the nuances and particulars of waiver applications to ensure that the proposed research meets the putative public interest criterion.^6^

A benefit of this approach is that it puts the onus on applicants to carve out the case for showing that their research sufficiently contributes to the public interest. But it introduces the possibility of inconsistency, incentivises researchers (and perhaps also IRB members) to inflate importance, and presupposes the competency and ability of institutions and IRBs to address the questions at hand.

#### Inconsistency

This approach requires careful judgment at two stages: institutions (or regulators) establishing a set of criteria that could justify a consent waiver on public interest grounds, then IRBs evaluating whether a given protocol meets those criteria. There are therefore two distinct opportunities for inconsistency in judgment to be introduced, and substantial scope for one IRB to come to a completely different judgment from another for the very same protocol – undermining claims to a system’s reliability and leaving it open to concerns about untrustworthiness.

#### Inflated promise

From a researcher’s perspective, waivers are very attractive – they open up datasets and tissue banks for use that would otherwise be restricted due to autonomy or privacy concerns. Conversely, rejecting a waiver could well mean a study must entirely be cancelled, or at least reworked, constituting substantial wastage of time and resources. Faced with such possibilities, applicants therefore might be incentivised to give an inflated report of various criteria – how important the research is, how many could benefit, its likelihood of success, etc.

#### Difficulty in evaluation

Perhaps a sufficiently well-trained IRB could see past such posturing, but that only raises a further question: are IRBs (as well as institutions and researchers) really equipped to make such judgment calls? Full evaluation of the impact of a research project is in the first place impossible until the results are produced. Projections may be made, but few researchers are equipped to do a full-scale rigorous impact assessment, complete with verifiable probabilities of success and ranges of outcomes on populations. This is especially the case for non-interventional research like secondary data or tissue studies. And even if projections were possible, it is not clear that IRBs would be in a position to evaluate them, or be disposed to challenge researchers’ assessments, especially the established ones. Left at this abstract level, appeals to the public interest might end up serving the same purpose as the need to show the prospect of (some) social value. As such, this would merely introduce redundancy into already burdensome bureaucratic processes.

#### Holistic evaluation of defined criteria

We contend that it is possible to build on a criteria-based framework, while at least mitigating some of the flaws. As noted, one of the main flaws of a criteria-based approach is the difficulty of making a consistent, accurate and broad assessment of a study’s contribution to the public interest. But criteria could be selected based on clarity, ease of formulation by researchers or IRBs, and general justifiability in terms of contributing to the public interest. Establishing a standardised array of information relating to the public interest criterion for the IRB to evaluate would limit divergence between judgments, at least at the IRB level, by ensuring all waiver applications are subjected to the same evidentiary basis of evaluation. Standardising evidence would also limit the ability of researchers to over-inflate impact by only providing information favourable to their case, and omitting information unfavourable. Finally, by setting well-defined criteria that all studies would have to meet, some burden on individual IRBs to determine what substantially contributes to the public interest and what does not would be limited.

Some examples of candidate criteria, along with a justification concerning contribution to the public interest, include addressing a health priority; scientific robustness; open access; non-patentability/copyright; and translatability. (See Table 2) We will not offer a thoroughgoing defence of why each of these criteria should be included in a holistic evaluation of the public interest criterion, as our intention here is not to specify the precise contents of such an evaluation. Instead, we offer these examples as illustrations of the sort of criteria we have in mind that could, when properly refined and justified, fill out a systematic evaluation of the public interest. Such examples could – alone or in combination – be set as threshold parameters for determining public interest in a given research context. For example, a research regulator wishing to promote research in a particular field might issue guidance that public interest will be more likely determined by robust scientific protocols that commit to open access, have a clear pathway for findings back to the clinic, and a defensible policy on intellectual property rights. We will return to some of these more procedural questions below.

**Table 2:** Candidate criteria for assessing the public interest in allowing consent waiver

Addressing a health priority	Many countries and institutions have identified top priorities in health or other areas. There are also the Sustainable Development Goals that have been set by the UN as key priorities for good health and well-being [23]. IRBs could rely on such a list, using it as a proxy for the areas of greatest public concern and favour waiver applications that address such areas – though being on this list would not be decisive in being granted a waiver. Rather, it would provide a good ethical reason in favour of waiver, setting the balance more towards that option.
Scientific robustness	All research should be scientifically valid in order to be approved, but studies may vary in terms of their robustness – that is, the extent to which the study as designed will be able to answer its research question(s) with a high degree of confidence. More robust studies will be more likely to contribute to generalisable knowledge, and thereby the public interest. Examples of factors contributing to robustness would be sample size, appropriateness of statistical methodology, and quality of the data. This would require independently verifiable evidence of such robustness to be submitted to any ethics decision-making body.
Open access of publications and datasets	As the primary output of biomedical research is knowledge that is meant to benefit future practice, allowing open access can help ensure such findings are indeed disseminated widely and have as much impact as possible [24]. This will require some extra expense on the part of research budgets, but should have the general social benefit of contributing to knowledge sharing. The recent move by several European funders to require publication of articles in open access journals that do not charge subscriptions might go some way towards ameliorating this difficulty, by putting pressure on an outdated model of high-cost academic publishing [25]. Such policies might also extend to deposits of raw data, after sufficient embargo periods to allow the primary researchers to publish from their own results.
Non-patentability/copyright of findings	While patents and copyright may serve the public interest by incentivising valuable research, they can also inhibit it by raising the cost of interventions and keeping those valuable insights out of the public domain. A research project committed to ensuring findings are kept on a creative commons or similar license would have a stronger case for contribution to the public interest than one that does not. Due to funding restrictions, not all valuable research may be able to meet this criterion, but the increased use of this criterion by ethics bodies might have the effect of putting pressure on funders to at least loosen or liberalise such license.
Translatability	Research whose results have direct, measurable relevance to practice or policy would in virtue of this have a greater claim to contribution to the public interest. For research that is more preliminary or concerned with proof of principle, evidence may instead be put forward of ‘clinical promise’: an explanation of how the research, in combination with other studies and evidence bases, could eventually lead to translational impact. This draws on the concept ‘clinical promise’ that has recently been urged by Kimmelman and Federico as a criterion for approval of first-in-human clinical trials [26].

This approach helps mitigate some of the challenges of open-ended criteria, by offering researchers and IRBs a structured set of definable components by which to evaluate a study. However, these criteria cannot provide on their own a formulaic or definitive answer to which studies meet a public interest criterion and which do not. Due to the nature of the concept of public interest, such a formula is unfortunately not possible, nor indeed is it desirable. Rather, this model provides non-exhaustive categories which *tend towards* contribution to the public interest that is supportive of a waiver, while at the same time allows decision-makers to expand and/or adapt the list on a case-by-case basis, thereby reflecting the criteria-based approach but still relative to this starting point of commonly-accepted elements that are indicative of advancing the health interest of the public or society.

At the end of the day, however, IRBs must still make a judgment, based on researchers’ responses to a series of queries, about whether a study sufficiently contributes to the public interest to merit a consent waiver. As such, further efforts are needed to ensure that the IRB’s judgments are consistent and justifiable. These matters are, in themselves, a matter of the public interest, helping to ensure that any model is knowable, fair, transparent and kept under regular review to respond to lessons learned from its operation in dealing with live health research applications.

### Procedural considerations in setting policy and practice

To meet this challenge of promoting consistency and justifiability, we propose adverting to procedural features of ethics review. Procedures do not themselves define which studies would fulfil the public interest criteria, but instead focus on helping ensure that the relevant decision-making mechanisms are fair, consistent and relevantly informed. Many of the procedures we outline below will not be unique to the issue of consent waivers, but we will show how applying them is particularly relevant and fruitful in this context. These procedures come in at two levels: setting a policy around the public interest criterion, and individual IRBs’ application of that policy.

#### Setting national or institutional policy

We previously highlighted several substantive approaches that may be taken to determining which studies meet a public interest criterion. At the very least, research institutions should set their own internal policies clarifying for IRBs and researchers not only how the determination of the public interest is to be undertaken, but also how in practice it should be evaluated.

Ideally, the overarching aim should be towards consistency in judgments concerning what studies meet the public interest criterion between IRBs and institutions within a given jurisdiction and over time. This may be achieved by a national initiative, which has the advantage of ensuring clarity of the core indicators and consistency with regulatory intent.

An alternative, if national policies are not forthcoming or if its definitions are still under-specified, is for institutions to promulgate their own practices and aim to harmonise them to a reasonable extent. Harmonisation may be difficult in many contexts due to difficulties in coordination between divergent institutions, but could be well worth the effort by minimising the possibility that an application for a waiver would succeed at one institution but fail at another. This would require mechanisms of communications and feedback between institutions, and we address this further below.

These policies would need to meet standard requirements of good governance. In particular, they should be transparent, reasonable and accountable [27]. Transparency of the policy (and its justifications) is crucial for adequate implementation, as well as allowing the policies to be open to legitimate scrutiny from concerned parties. Reasonableness is understood as being justifiable to stakeholders regardless of their personal parochial views; any accepted notion of the public interest must in this way appeal to values that are, in turn, of appeal to the general population, and not just a subset. And accountability is necessary to make clear whose job it is to establish and evaluate the public interest of research for any given application. IRBs will presumably have a central role, but researchers must assume responsibility for their study and make all reasonable efforts to identify and justify the public interests and goods they seek to advance. This could be done with the support of other bodies like funding agencies, sponsors, institutions and regulators.

Perhaps most importantly, the process of delineating the public interest criterion should be done in consultation with the public (or ‘publics’) at large. Suggestions are offered in this paper and elsewhere^3^ on how this can be done, but a procedurally robust approach would not simply adopt the arguments from academic literature. Because public interest, by definition, appeals to the sensibilities and the needs of the public – and in recognition that these matters will change over time with social mores and values and exposure to the benefits of research efficiency - the final conceptualisation of public interest adopted by institutions must incorporate public attitudes and values, or at least be able to give a robust account relative to the same. The pragmatics of such public engagement are outside the scope of this paper, but some models can be highlighted: citizens’ juries; community consultation and community representation.

A citizens’ jury comprised of a random selection of individuals that are demographically representative of the general public could deliberate on the question of what research counts as being in the public interest [28]. Jurors would receive ‘testimony’ and evidence, but it would ultimately be up to them to decide (as a jury would) what answer should be accepted. Such approaches are especially useful in cases like the present, where the complexities of the question make a traditional survey or focus group inadequate to ensure deliberation is adequately informed. However, there are limitations of citizens’ juries that should be noted, including bias and advocacy hijacking [29].

More broadly, potential policy options should be subject to a process of consultation (and, ideally, co-production) with key community and stakeholder groups [30]. Researchers themselves will be an important group to consult, especially those who will be seeking the consent waivers. Defining the public interest in the abstract may not be possible in such a forum, so a case-based approach may be preferred: propose example cases, and interrogate the extent to which they are deemed sufficiently in the public interest to merit a consent waiver. Analysis could then be performed to determine patterns behind which cases were accepted and which were not. And those initial, agreed-upon paradigmatic cases could be used as a resource and reference guide by IRBs as well as researchers for future applications. The precise shape of the consultation will depend on the social context and practical resources available.

Separate from community consultation, it would be advisable for the policymaking body that determines how public interest should be defined to contain members of the lay public – individuals not themselves involved in research or research oversight. This is to ensure that the final results are indeed reasonable for including a spectrum of views, in the sense that they are based not just on the parochial values of an institution or other body setting policy, but incorporate or take into account values held by the community at large as well.

#### IRB review

Procedures adopted by IRBs would need several safeguards to promote consistency and reasonableness. The following proposals might be adopted at a systems level, across a jurisdiction, if a more top-down approach is taken; or they could be taken up by individual IRBs and/or their institutions if such systems-level coordination is not present.

At least early on in implementation, such waiver applications require subjective judgment and, as such, should be reviewed by a full board. This may be more administratively onerous, but it is essential because of the nature of expedited or exempt reviews: they both involve review by perhaps one or two IRB members. This increases the risk of arbitrary interpretations or individual members’ idiosyncratic values affecting judgment and leading to inconsistency of judgment between decision-making bodies. A full board would still have to make a judgment, but by coming to a consensus such arbitrariness can at least be mitigated. Over time, as consistent, precedent-based understanding of what qualifies as in the public interest would be established (see the next subsection), the risk of arbitrariness will go down; at that point, it may be possible to relax this requirement.

Relatedly, IRBs should ensure that (as with the policy-making body), the perspectives of the lay public are adequately captured, as relevant to a given proposal. Again, this is to ensure that institution-specific values and perspectives do not dominate the judgment being made. Lay membership is generally seen as a requirement for IRBs [31], but it is especially important (and representation therefore may need to be greater) for subjective evaluations of the public interest. While scientific members of an IRB provide expertise essential to understanding a study’s risks and benefits, scientific robustness, and other important features of review, such expertise will be much less central to assessing whether the study substantially advances the public interest. Instead, having a broader array of perspectives on the value of a given study will help avoid ethical blind spots (e.g., overlooking or underemphasising the interests of some subgroups) and enrich the evaluation of the public interest criterion.

The reasons for granting a waiver, or especially for rejecting it, should also be made transparent to the researcher. The researcher may (reasonably) wish to appeal an unjustified rejection, or else alter the study to improve its prospect for contributing to the public interest and re-submit accordingly. It will also help promote trust in the approval system, warding it against the charge that its decisions are arbitrary, as well as prompt reasoned critique in the system that could be used by IRBs and institutions to improve their approach. Moreover, aggregate reasons for granting and rejecting waivers should, in due course, be made public both in the interests of transparency as well as to increase learning from the system. This should not include researcher-level information, merely classes of research and types applications and brief accounts of reasons for the decisions in interpreting the public interest criterion.

These additional procedures are especially important if criteria are used in evaluating public interest. The subjective nature of such judgments leads to the challenges discussed earlier on, but these procedures may go some ways towards addressing those challenges.

#### Periodic review

Neither policies nor IRB practice should not be considered static, but should be subject to periodic review to ensure that practices are indeed meeting expectations. In particular, has the approach reached an adequate and acceptable balance between pragmatic operability and ethical justifiability? This review need not be in the form of top-down governmental audit, but could be internal or inter-institutional.

This systems review can address inconsistency of judgments between and even within IRBs, one of the more glaring difficulties in evaluating public interest. Review should compile summaries of a series of cases that have been adjudicated, with two purposes in mind.

First, within a given institution, precedent can be established. Just as was suggested above for hypothetical cases at the policy-setting stage. Institutions can thereby derive patterns and norms from the judgments that have been made. These judgments can first and foremost be evaluated to ensure they do, in fact, meet a reasonable standard of ethical justifiability. They can, moreover, provide a useful guide to future IRBs as well as researchers to facilitate understanding of which sorts of studies pass the bar, and which do not. This can ensure that IRB member turnover and review by different members does not substantially affect the outcome. Such a model was adopted by the Confidentiality Advisory Group in England and Wales for use of medical records [32], and this could be replicated elsewhere.

Second, both between and within institutions, cases can be used to determine whether or not IRBs are consistent with one another in their evaluative approach. Is one IRB (or IRB member) evaluating at a much higher bar than another? If so, this is a problem that would need to be addressed, ideally by the inconsistent parties meeting, discussing their differences, and working towards a mutual understanding to reduce such inconsistencies in the future. This might also lead to system-wide (re)training for IRBs, all learning from the experiences of the few. For this purpose, something like the UK’s Shared Ethical Debate (ShED) tool could be used. Under the ShED model, identical protocols are sent out to multiple ethics committees, whose evaluations are then compared for consistency. Current ShED results show that even in more well-defined areas of ethics review, there is substantial inconsistency. The inconsistency has, however, been reduced over time, owing in part to feedback given to ethics committees regarding the extent to which their responses align with other committees’ [33]. This leverages committees’ intrinsic motivation to seek inter-committee consistency by highlighting to outlier committees how their judgments may be more aberrant than they realize. A reduction in inconsistency is especially important for evaluating public interest claims, as interpretation is likely to foster substantial disagreement.

Such review processes would be much more straightforward in systems with national coordination of IRBs. In jurisdictions lacking a national system, it will instead be up to the IRBs to take it upon themselves to come together for such a review exercise. While this may be time-consuming, it will help promote the legitimacy and reliability of decision-making within the jurisdiction. Indeed, such review need not be limited to evaluating the public interest criterion – both the ShED and Confidentiality Advisory Group examples above are wider in scope.

Whatever the institutional or national set-up, however, the key point here is that some mechanism of mutual learning ought to emerge from (within) ethics decision-making bodies when it comes to interpretations and applications of the public interest criterion. In the very least, adopting this approach could improve the consistency in evaluating the public interest criterion both between members of a given IRB, and between different IRBs. More broadly, it could also help improve confidence towards and trust in the ethics review process amongst researchers as well as the general public. By establishing not only a set of clear, reasoned criteria for meeting the public interest requirement, but also a system of evaluation and refinement of the relevant standards, institutions and IRBs can demonstrate responsiveness and flexibility in governance of human subjects research.

## Conclusion

In this paper, we have striven to offer an understanding of the public interest criterion for the purpose of consent waivers, and offered some suggestions as to the best way to implement such a requirement. We have proposed an approach that analyses how (i) public interest can best be understood in the present context as a justification for research using data/samples; (ii) well-defined criteria may be useful for IRBs/researchers to determine whether a given study meets the criterion; (iii) existing good governance frameworks to the second-order considerations for procedural measures concerning policies/institutional practices related to public interest can be applied, and (iv) application of the public interest criterion should be reflexive, using measures such as periodic intra-IRB review. This approach balances the need to be sensitive to different studies’ nuances with the need to be efficient, transparent, accountable and consistent across domains. And we have also suggested the procedural features that should be met when determining what counts as contribution to the public interest. This is not meant to be a decisive determination, as actual practice will require adaptation to institutional context, resources, and capacity. But we hope it can serve as a useful guide to institutions and IRBs struggling with the difficult question of how to evaluate which studies may indeed be contributing to the public interest.

## Data Availability

Not applicable

## Abbreviation

IRB: Institutional review board

## Footnotes

1 Here, ‘personal data’ is understood as identifiable data about a person. Data may be identifiable if the dataset contains unique personal identifiers, or if someone in conjunction with other data to which they have access could reasonably re-identify individuals within it.

2 We use the term ‘IRB’ for convenience, recognising in some jurisdictions equivalent bodies are referred to as research ethics committees or similar.

3 Ballantyne, A, Schaefer, GO. Public interest in health data research: laying out the conceptual groundwork. Unpublished manuscript currently under review

4 For the purpose of this paper, we remain neutral on whether social value is essentially the same concept as public interest, or a distinct notion. For an argument that they are distinct, see footnote 3.

5 That is not to say all research benefitting children would necessarily be sufficiently contributing to the public interest to merit a waiver of parental consent.

6 This is roughly the approach put forward by the Ministry of Health in Singapore; see its ‘Guidance on the Requirement of Appropriate Consent for the Conduct of Human Biomedical Research and Handling of Human Tissue’ [22].
